# Comparison of Methods for the Reconstruction of the Hepatic Artery in Mouse Orthotopic Liver Transplantation

**DOI:** 10.1371/journal.pone.0133030

**Published:** 2015-07-24

**Authors:** Ning Pan, Zhenzhen Liu, Jinjing He, Song Li, Xiangwei Lv, Liming Wang, Qinlong Liu

**Affiliations:** 1 Department of Anesthesiology, The Second Affiliated Hospital of Dalian Medical University, Dalian, Liaoning Province, China; 2 Department of General Surgery, The Second Affiliated Hospital of Dalian Medical University, Dalian, Liaoning Province, China; 3 Dalian Medical University, Dalian, Liaoning Province, China; University of Colorado Denver, UNITED STATES

## Abstract

**Background:**

The mouse model of arterialized orthotopic liver transplantation (AOLT) has played an important role in biomedical research. The available methods of sutured anastomosis for reconstruction of the hepatic artery are complicated, resulting in a high incidence of complications and failure. Therefore, we developed and evaluated a new model of AOLT in mice.

**Materials and methods:**

Male inbred C57BL/6 mice were used in this study. A continuous suture approach was applied to connect the suprahepatic inferior vena cava (SHVC). The portal vein and infrahepatic inferior vena cava (IHVC) were connected according to the "two-cuff" method. The common bile duct was connected by a biliary stent. We used the stent (G3 group) or aortic trunk (G2 group) to reconstruct the hepatic artery. The patency of the hepatic artery was verified by transecting the artery near the graft after one week. The survival rate of the recipients and serum alanine aminotransferase (ALT) levels, hepatic pathologic alterations, apoptosis and necrosis were observed at one week postoperatively.

**Results:**

The patency of the hepatic artery was verified in eight of ten mice in G3 and in six of ten mice in G2. The 7-day survival rate, extents of necrosis and apoptosis, and TGF-β levels were not significantly different among the three groups (P>0.05). However, the serum ALT levels and operation time were markedly lower in G3 compared with G2 or G1 (both P<0.05).

**Conclusions:**

Reconstruction of the hepatic artery using a stent can be performed quickly with a high rate of patency. This model simplifies hepatic artery anastomosis and should be promoted in the field of biomedical research.

## Introduction

Hepatocellular carcinoma (HCC) is the sixth most common carcinoma worldwide and is one of the leading causes of cancer-related death [1]. Orthotopic liver transplantation (OLT) continues to be the standard and classic principle in the curative treatment of HCC [2]. The rapid development of research investigating liver transplantation has benefited from a variety of experiments in small and large animals. Rodent models of liver transplantation play an extremely important role in biomedical research. In 1973, Lee et al. introduced for the first time a rat model of OLT [3]. This model involved vascular reconstruction by the suture technique, which is extremely complicated and causes anhepatic phase extension. In 1979, Kamada and Calne modified the approach of vascular reconstruction by using a polyethylene cuff for anastomosis of the portal vein and the infra-hepatic vena cava. The cuff method shortened the anhepatic time of graft implantation, improved the success rate of the operation and reduced the learning curve. Progress in genomic technologies has resulted in a mouse model of OLT that is physiologically and clinically more similar to human OLT when compared with rat or other rodent models. In 1991, based on Kamada’s cuff approach, Qian et al introduced a mouse model of OLT in which arterial reconstruction was not performed, resulting in a short duration of mouse survival [4, 5]. Because of the technical difficulties associated with the smaller size of the mice, fewer modifications of the mouse AOLT model have been developed compared with the rat AOLT model. In 2002, however, Tian et al used an end-to-side suture anastomosis method between the donor superior mesenteric artery and the recipient abdominal aorta to reconstruct the hepatic artery and to demonstrate that arterial reconstruction of OLT is critical for long-term survival [6]. The end-to-side suture anastomosis for reconstructing the hepatic artery has been the classical technique in the mouse model of AOLT. Because the procedure is extremely complex, it requires additional time, and the longer arterial segment is more inclined to kink and lead to a subsequent thrombosis. These characteristics result in a high incidence of complications and failure. In 2014, Zhou et al used Micro-Renathane implantation tubing as the arterial stent to reconstruct the hepatic artery and demonstrated that this procedure could be performed quickly and with a high rate of patency [7]. However, Micro-Renathane implantation tubing is costly, and the difference between reconstruction of the hepatic artery by stent compared with end-to-side suture anastomosis for hepatic artery reconstruction by the aortic trunk remains unknown. The purpose of this report is to evaluate the use of the common polyurethane stent to reconstruct the hepatic artery in mice and elucidate the difference between the reconstruction of the hepatic artery by stent and by the aortic trunk.

## Materials and Methods

### Animals and Study Design

#### Ethic Statement

This study was carried out in strict accordance with the commendations in the Guide for the Care and Use of Laboratory Animals of the National Institutes of Health. The protocol was approved by the Committee on the Ethics of Animal Experiments of the Dalian Medical University, China (Permit Number: SYXK (Liao) 2008–0002). All surgery was performed under isoflurane anesthesia, and every effort was made to minimize suffering. After transplantation, all of the recipients were maintained in a temperature-controlled environment with a 12-h light—dark cycle with free access to standard laboratory chow and tap water. Fentanyl was used 24 h after surgery. The condition of the mice was monitored every hour in the daytime and every 6 h at night. Mice were under isoflurane anesthesia again before collecting blood and liver samples for clinical chemistry, histology and immunohistochemistry, and then sacrificed by cervical dislocation under anesthesia. For survival studies, mice in markedly poor condition (poor condition of signs are ruffled fur, hunched posture, lethargy) were considered to have failed to survive from transplantation. These mice were then put into coma with carbon dioxide and humanely euthanized by cervical dislocation. Mice which had lived for more than 7 days after transplantation were considered survivors.

#### Animals

Male inbred C57BL/6 mice, aged 10–12 weeks and weighing between 27 and 30 g (purchased from the Animal Center of Chongqing University of Medical Sciences, China), were housed in a standard animal laboratory with free activity and access to food and water, and kept under constant environmental conditions with a 12:12-h light: dark cycle.

#### Study Design

Mice were randomly assigned to an experimental group or a sham group. The mice in the experimental group were classified either as donors or recipients and randomly divided into three groups: without reconstruction of the hepatic artery (G1), reconstruction of the hepatic artery by the aortic trunk (G2), and reconstruction of the hepatic artery by the stent (G3). The weight of a recipient was equal to or slightly more than (no more than 5 g) that of the donor. All of the mice were fasted for 12 h before the operation without water deprivation. All of the surgical procedures were performed under isoflurane anesthesia, and every effort was made to minimize suffering. The grafts were stored in University of Wisconsin (UW) solution at 4°C for 6–8 h. The survival rate of the recipients was monitored at one week, at which time all of the mice were killed. The mice were anesthetized with isoflurane before the collection of blood and liver samples and then sacrificed under anesthesia by cervical dislocation. All of the surgical procedures were performed under isoflurane anesthesia, and every effort was made to minimize suffering. Serum alanine aminotransferase, hepatic pathologic alterations, apoptosis and necrosis were observed at one week postoperatively. Survival was monitored for 7 days following surgery. Mice that had lived for more than 7 days after transplantation were considered survivors.

### Surgical procedure

All of the surgical procedures were performed under aseptic conditions using a binocular microscope with the aid of 6-40x magnification objective lenses (Shanghai Medical Instrument Factory, China). Isoflurane was administered as a general inhalation anesthetic in all cases. The liver grafts were stored in UW solution at 4°C for 6–8 h, and a continuous suture approach was used to connect the suprahepatic inferior vena cava (SHVC). The portal vein and the infrahepatic inferior vena cava (IHVC) procedure was based on the "two-cuff" method. The common bile duct was connected by a biliary stent. The hepatic artery was not reconstructed in G1. In G2, the hepatic artery was reconstructed using the aortic trunk method. In G3, the hepatic artery was reconstructed by stent.

#### Donor operation

Isoflurane inhalation was used to anesthetize the mice. The prospective operational area of the anesthetized mice was shaved, and donors were immobilized on the operating table. Subsequently, the abdomen was disinfected with 75% isopropyl alcohol. A wide cross incision was used to open the abdomen. The xiphoid was retracted with a clamp to improve exposure of the upper abdomen. To reveal the IHVC and hepatic hilus, the intestines were moved to the left and protected in moist saline-soaked gauze. Next, 50 IU heparin was injected via the IHVC with a 30-gauge needle. The dissection was initiated by freeing the ligamentous attachments of the liver from its surrounding organs. The branches of the esophageal plexus running toward the liver were then diathermied and divided. The falciform ligament was subsequently divided completely, and the left phrenic vein was coagulated and divided. Next, the IHVC was skeletonized completely, and then the cystic duct was ligated and the gallbladder removed along the gallbladder bed. Subsequently, the duodenum adjoining the common bile duct was beveled and the bile duct of the donor was cannulated with a 1-cm-long, stretchable, disposable epidural catheter, which was inserted into the distal bile duct lumen and secured with a circumferential 7–0 silk suture. The gastroduodenal artery was mobilized and dissected from the common hepatic artery, and the splenic artery and left gastric artery were subsequently ligated and blunt-dissected along the celiac trunk and then isolated from the abdominal aorta. The lumbar arteries were separated from the aorta and successively ligated. The proximal PV was tied off, and a transverse incision was made on the anterior wall of the PV near the tie, through which an intravenous cannula (18 gauge) was inserted to perfuse the liver with 1 ml of solution at 4°C. The IHVC was incised at the level of the right renal vein to release the blood. At the end of the perfusion, the liver was blood free and uniformly turned yellowish gray. The SHVC was then transected at the level of the diaphragm. The PV was divided at the level of the incision point. Physiological saline (4°C) was continuously showered on the liver to maintain it at a low temperature. The abdominal aorta above the celiac trunk was then blunt dissected, and a 7–0 silk was placed on the opposite portion of the abdominal aorta and ligature of the celiac trunk. The superior mesenteric artery, right renal artery, left renal artery and lumbar arteries were separated and ligated successively, and the aorta was transected above the celiac axis near the level of the iliac artery. Thus, the hepatic—celiac axis—aortic artery segment was carefully preserved. The liver was carefully immersed in solution at 4°C for cuff preparation.

#### Cuff preparation

**Fig 1:**
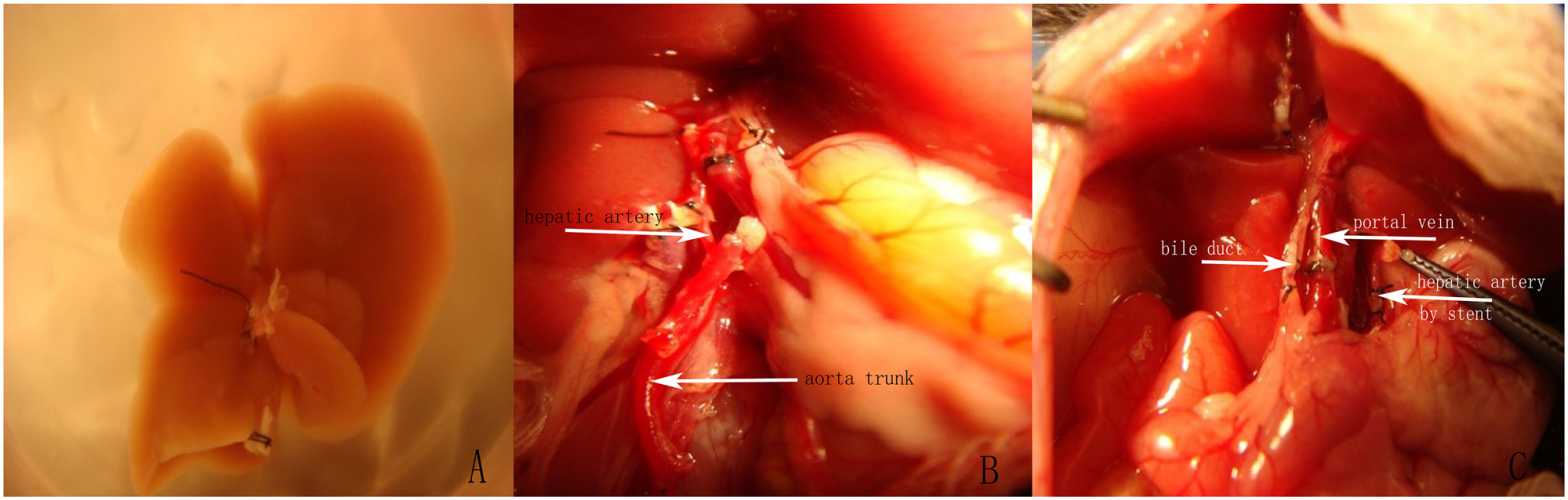
Pictures in the operation. (A) The liver graft was stored in UW solution at 4°C. (B) Reconstruction of the hepatic artery by aortic trunk group (G2). (C) Reconstruction of the hepatic artery by stent group (G3).

The cuff preparation was performed in a bath container filled with UW solution on ice. The 1.5-mm-long venous cuffs were prepared from 20-gauge intravenous cannulas for IVC and 18-gauge intravenous cannulas for PV (length: 1.5 mm; PV cuff: 18-gauge intravenous cannulas; inner diameter: 0.9 mm; outer diameter: 1.3 mm; IHVC cuff: 20-gauge intravenous cannulas; inner diameter: 1.2 mm; outer diameter: 1.7 mm). The PV was dissected free from the bile duct and debrided. The cuff extension was held with forceps, and another forceps was passed through the lumen of the cuff tube to grasp the PV. The cuff was then slipped over the PV. Next, the cuff extension (without the PV) was secured with a forceps, which was fixed to the wall of the bath container. At this point, the open end of the PV was spread with two pincettes. The end was everted over the cuff body and secured in this position with a circumferential 7–0 silk suture. The same method was applied to the IVC. After preparation, the liver was again perfused via the PV and IVC with 1 ml of 4°C solution to ensure that twisting of the PV and IVC within the cuffs did not occur. Subsequently, the livers were stored in UW solution at 4°C, as shown in Fig 1A.

#### Recipient operation

Isoflurane inhalation was used to anesthetize the mice. A midline abdominal incision was generated, and the xiphoid process was elevated with a clamp. The liver was separated in a manner similar to that of the donor. The ligamentous attachments of the liver were divided. Both the right suprarenal vein and the lumbar vein were ligated and divided. The left phrenic vein and branches of the esophageal plexus were ligated and divided. The hepatic artery and common bile duct were divided close to the liver hilum. The aorta under the left renal artery was mobilized, and all of the branches were divided and separated. The PV and IHVC were cross-clamped with microvessel clips, and the SHVC was cross-clamped with a Satinsky clamp. The vessels were then divided close to the liver. The PV and IVC were left as long as possible to facilitate cuff anastomosis. The recipient liver was removed. After being rinsed with 1 ml of saline solution via PV, the donor liver was removed from the 4°C bath, placed in the orthotopic position, and covered with a wet swab. First, the donor SHVC was anastomosed end to end with the recipient SHVC using a running 10–0 Prolene suture. Then, the PV anastomosis commenced. The cuff of the donor PV was inserted into the PV lumen of the recipient. To prevent an air embolism, the anastomotic site was washed out with saline solution. The anastomosis was completed with a circumferential 7–0 silk suture. Using the same method, the IHVC was connected. Subsequently, the clamps on the PV, SHVC and IVC were released. Reconstruction of the hepatic artery was used for the hepatic—celiac axis—aortic segment, which was anastomosed to the recipient aorta with 10–0 Prolene in G2, as shown in Fig 1B. A segment of the abdominal aorta below the left renal artery, prepared as described above, was blocked at the proximal and distal ends of the artery clamp. An opening was generated in this section of the abdominal aorta of the recipient to anastomose the abdominal aorta of the donor via "end-to-side anastomosis". When the artery clamps at the proximal and distal ends were loosened, the hepatic artery quickly filled with blood. An oblique opening was created on the anterior wall of the common hepatic artery with micro-scissors, through which the stent connecting the arterial segment was inserted and secured with a 7–0 silk suture fixed from both ends in G3, as shown in Fig 1C. The bile duct was anastomosed by inserting the splint and secured with a 7–0 silk suture. The abdominal incision was closed in two layers with running 4–0 Vicryl and 4–0 Prolene sutures. After transplantation, all of the recipients were warmed with a heating pad and had free access to standard laboratory chow and tap water.

### Clinical chemistry, histology and immunohistochemistry

Blood was collected from the IVC, and the livers were recovered. Serum alanine transaminase (ALT) was measured using analytical kits from Pointe Scientific (Uncoln Park, MI) [8]. Histology was performed using hematoxylin-eosin (H&E) staining. Necrotic areas were quantified in a blinded manner by image analysis of 10 randomly selected fields per liver in slides stained with H&E using a IPlab 3.7v software (BD Biosciences, Rockville, MD) [9]. Apoptosis was assessed by terminal deoxynucleotidyl transferase dUTP nick-end labeling (TUNEL) using an In Situ Cell Death Detection Kit [10]. RT-PCR was used to determine the expression of transforming growth factor-β (TGF-β) mRNA.

### Statistical analysis

Data are presented as the mean ± SEM. The groups were compared using the Kaplan—Meier test and ANOVA plus the Student—Newman—Keuls (S-N-K) or Fisher's least significant difference test (LSD) as appropriate. There were 10 mice per group in the survival experiment and 6 per group for all of the other experiments, as indicated in the figure legends. Differences were considered significant at p < 0.05.

## Results

### Duration of surgery

**Fig 2:**
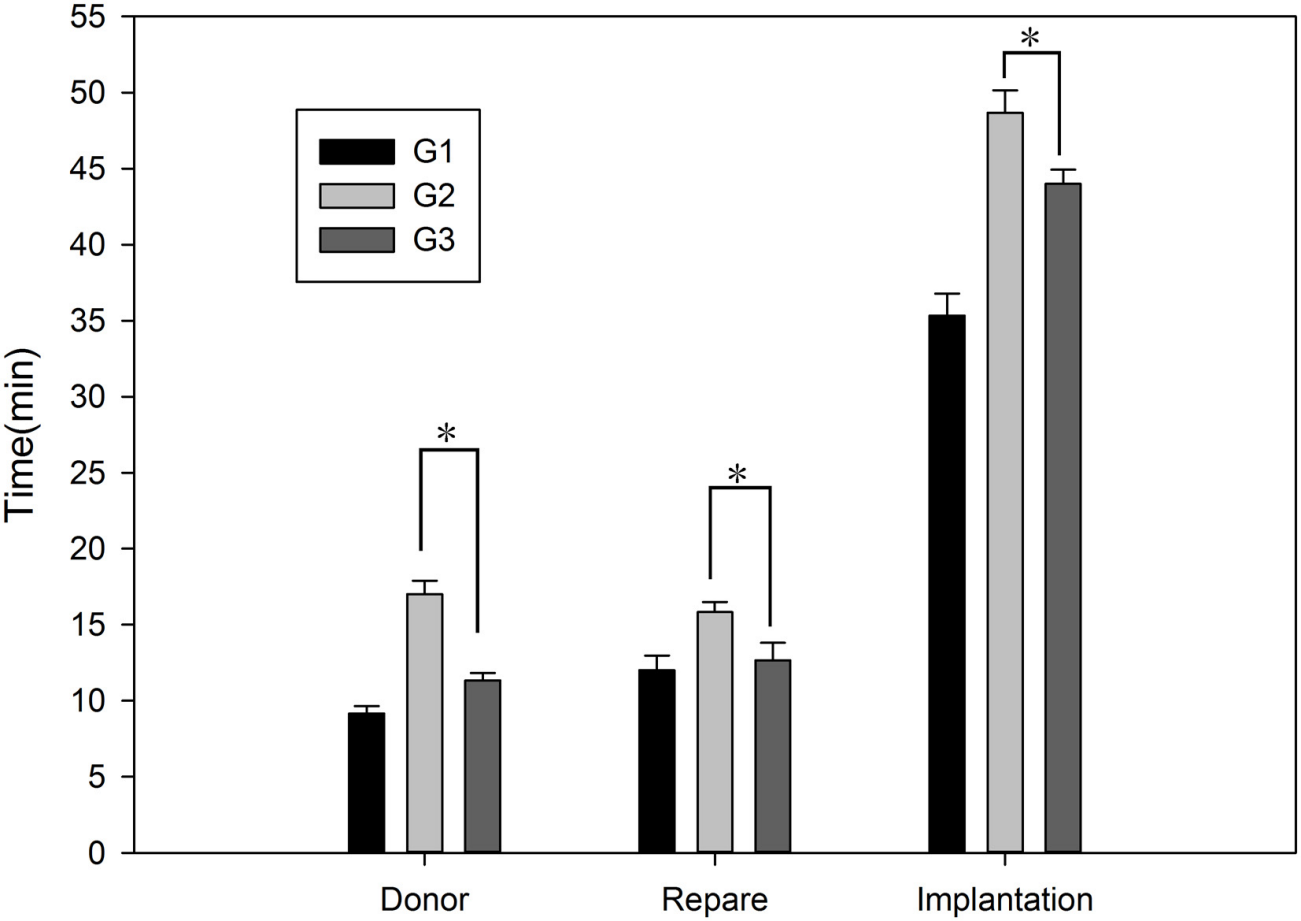
The mean times of donor operation, repair, and implantation in each group. The means + SEM of six mice are shown.

**Fig 3:**
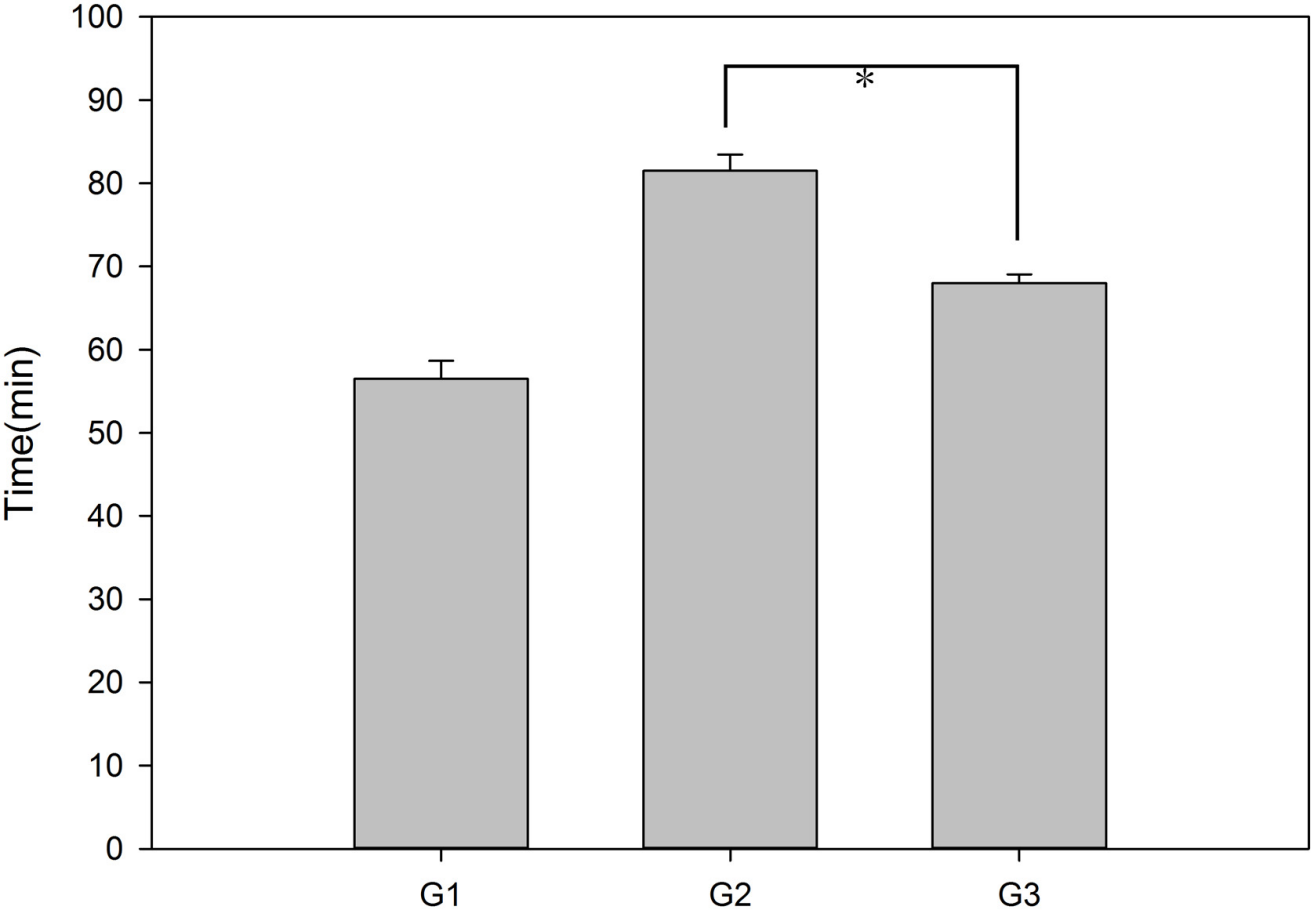
The mean total operation time in each group. The means + SEM of six mice are shown.

The grafts were stored in UW solution at 4°C for 6–8 h, and the recipients underwent OLT using a two-cuff technique. The mean implantation operation time in each group was 35.3 min (G1), 48.6 min (G2), and 44.0 min (G3), with a significant difference (p = 0.000) among the three groups and between G3 and G2 (p = 0.024). The mean operation time for the donor in each group was 9.2 min (G1), 17.0 min (G2), and 11.3 min (G3), demonstrating a significant difference (p = 0.000). The mean repair time in each group was 12.0 min (G1), 15.8 min (G2), and 12.7 min (G3), revealing a significant difference (p = 0.026), as shown in Fig 2. The mean total operation time in each group was 56.5 min (G1), 81.5 min (G2), and 68.0 min (G3), which was significantly different (p = 0.000), as shown in Fig 3. The reconstruction of the hepatic artery by stent can save approximately 4.6 min compared with the aortic trunk method. All of the recipients survived during the time required for the hepatic artery reconstruction, which ranged from 2–15 min. The mean time needed for reconstruction of the hepatic artery was 3 min in G3, whereas it was 6 min in G2.

### Patency and survival rate

**Fig 4:**
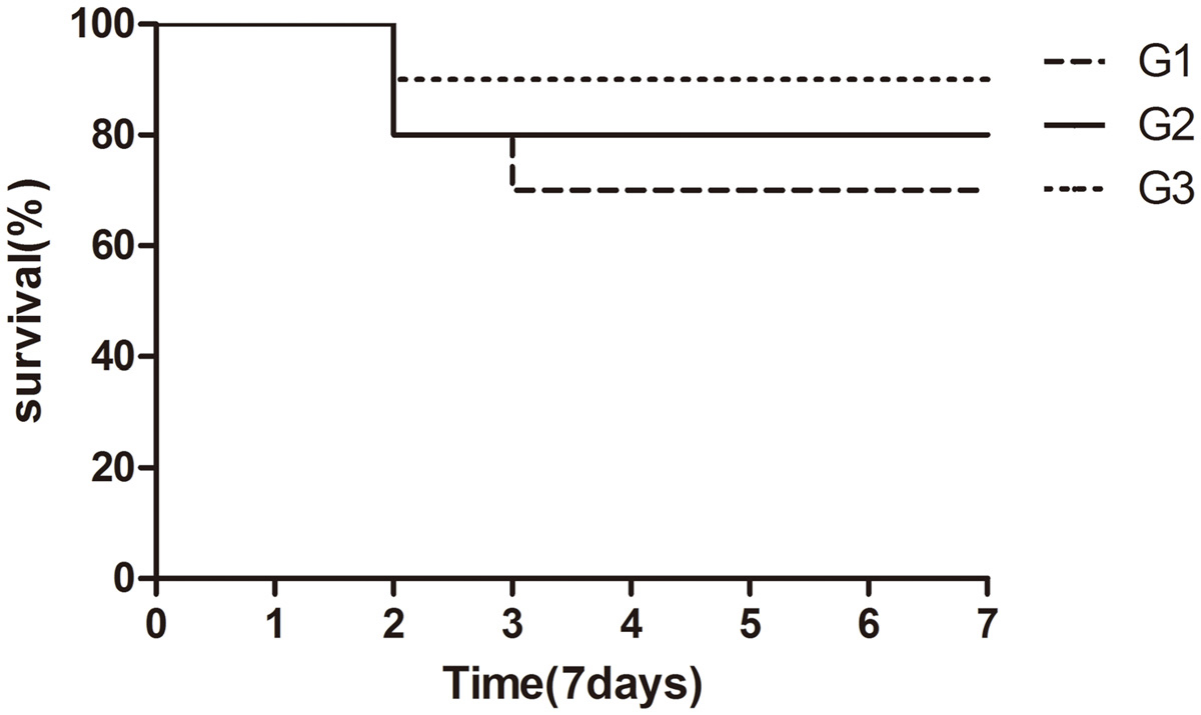
The 7-day survival rate after liver transplantation. Mice were observed for 7 days postoperatively. There were 10 animals in each group. The 7-day survival rate in each group was 90% (G3), 80% (G2), and 70% (G1) according to the Kaplan-Meier test (P>0.05).

The patency of the hepatic artery was verified by transecting the artery near the graft after one week. The patency of the hepatic artery was verified in eight of ten mice in G3 and in six of ten mice in G2. There were no significant differences in the 7-day survival rate among the three groups (p = 0.0557), as shown in Fig 4. The 7-day survival rate in each group was 90% (G3), 80% (G2), and 70% (G1). An increased number of mouse deaths occurred during the 2nd to 3rd day after the operation. The reason for death was likely acute diffuse peritonitis.

### Serum alanine aminotransferase (ALT)

**Fig 5:**
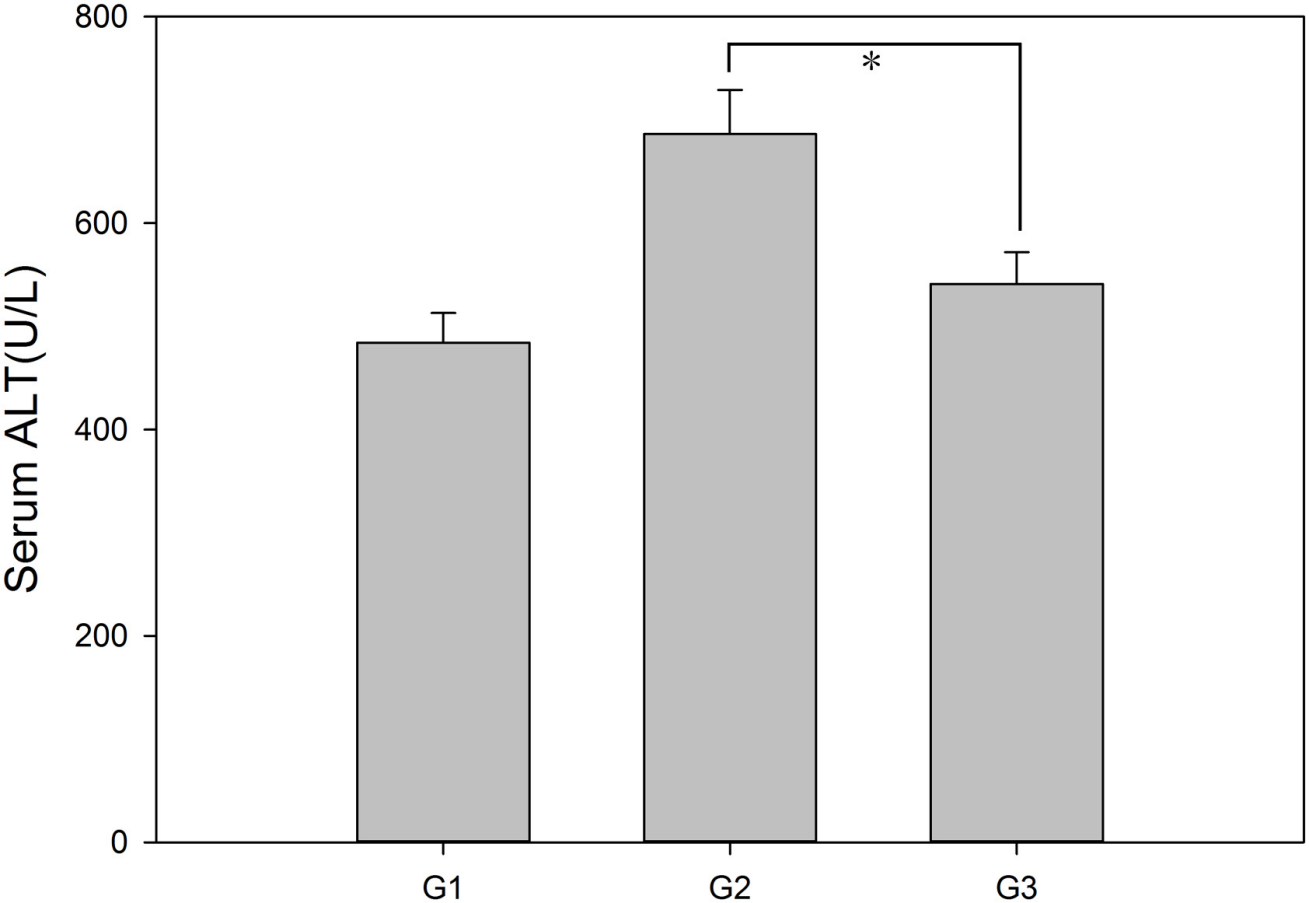
ALT levels in mice at 7 days after transplantation. Before the mice were sacrificed for histology, blood was collected to determine the levels of serum alanine aminotransferase. The means + SEM of six mice are shown.

The levels of serum alanine aminotransferase (p = 0.03), a sensitive marker for evaluating the extent of hepatocellular injury [11], were markedly lower in G1 compared with G2 or G3. The mean serum ALT levels in each group were 484.3333 ± 28.5524 U/l (G1), 686.3333 ± 42.6291 U/l (G2), and 541.1667 ± 30.7250 U/l (G3), as shown in Fig 5. However, there were no obvious differences in serum ALT levels between G2 and G3 (p = 0.09). The mean serum ALT level was higher in G2 than in G3.

### Histopathology

**Fig 6:**
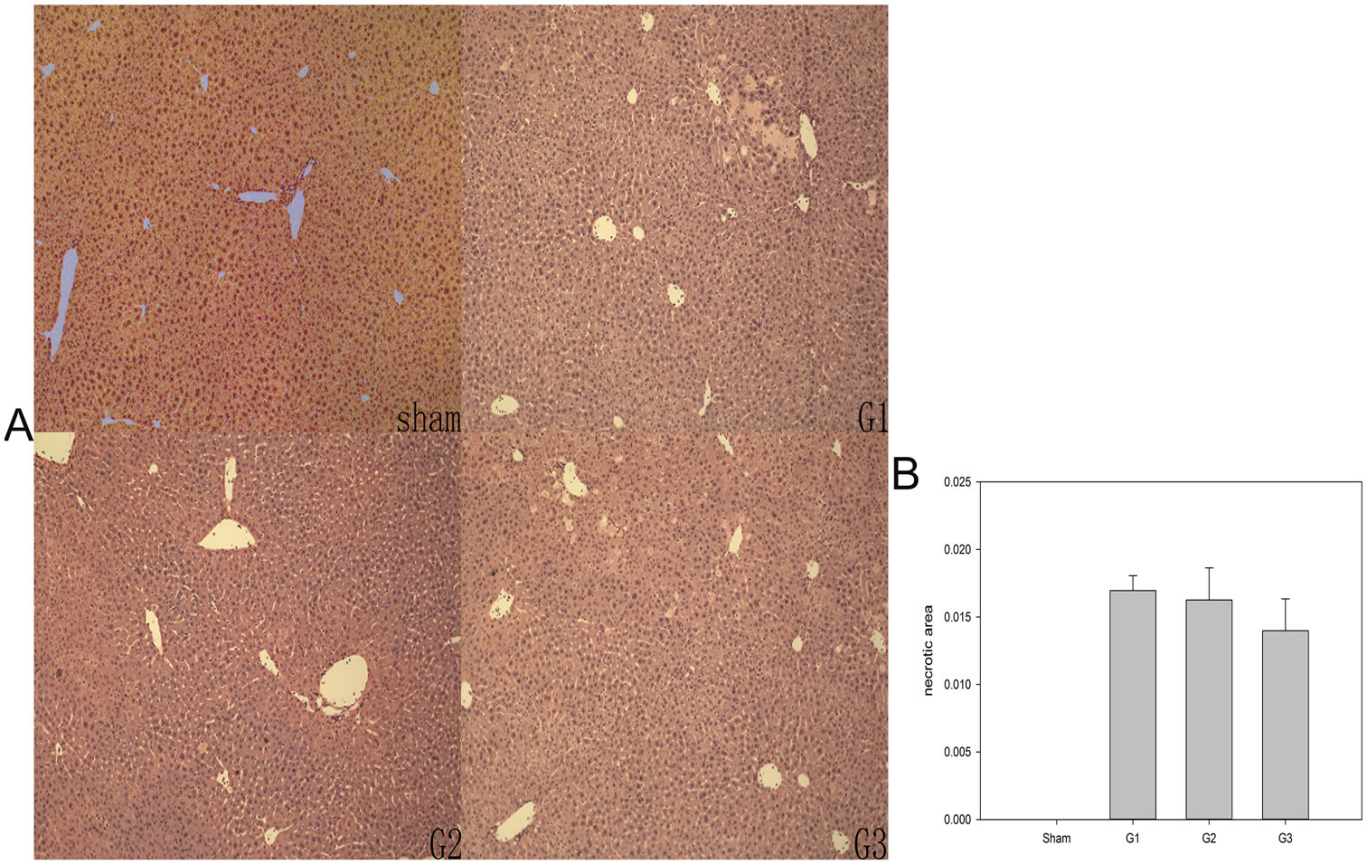
(A) Histology of the graft after 7 days of OLT. Hematoxylin-eosin-stained liver section from the grafts of each group at 7 days after transplantation showing small foci of necrosis (magnification 200×). (B) The necrotic areas of the graft after 7 days of OLT. The necrotic areas were quantified in a blinded manner by image analysis of 10 randomly selected fields per liver in slides stained with hematoxylin-eosin using IPlab 3.7v software (BD Biosciences, Rockville, MD). The means + SEM of six mice are shown.

In the sham group, no hepatic histopathological changes were observed after H&E staining (Fig 6A). Necrosis was apparent in G1, G2, and G3 (Fig 6A). The area evaluated for necrosis displayed no marked differences (p = 0.522) among the three groups. However, the area of necrosis in G3 was smaller than that in G2 and G1, as shown in Fig 6.

### TGF-β

**Fig 7:**
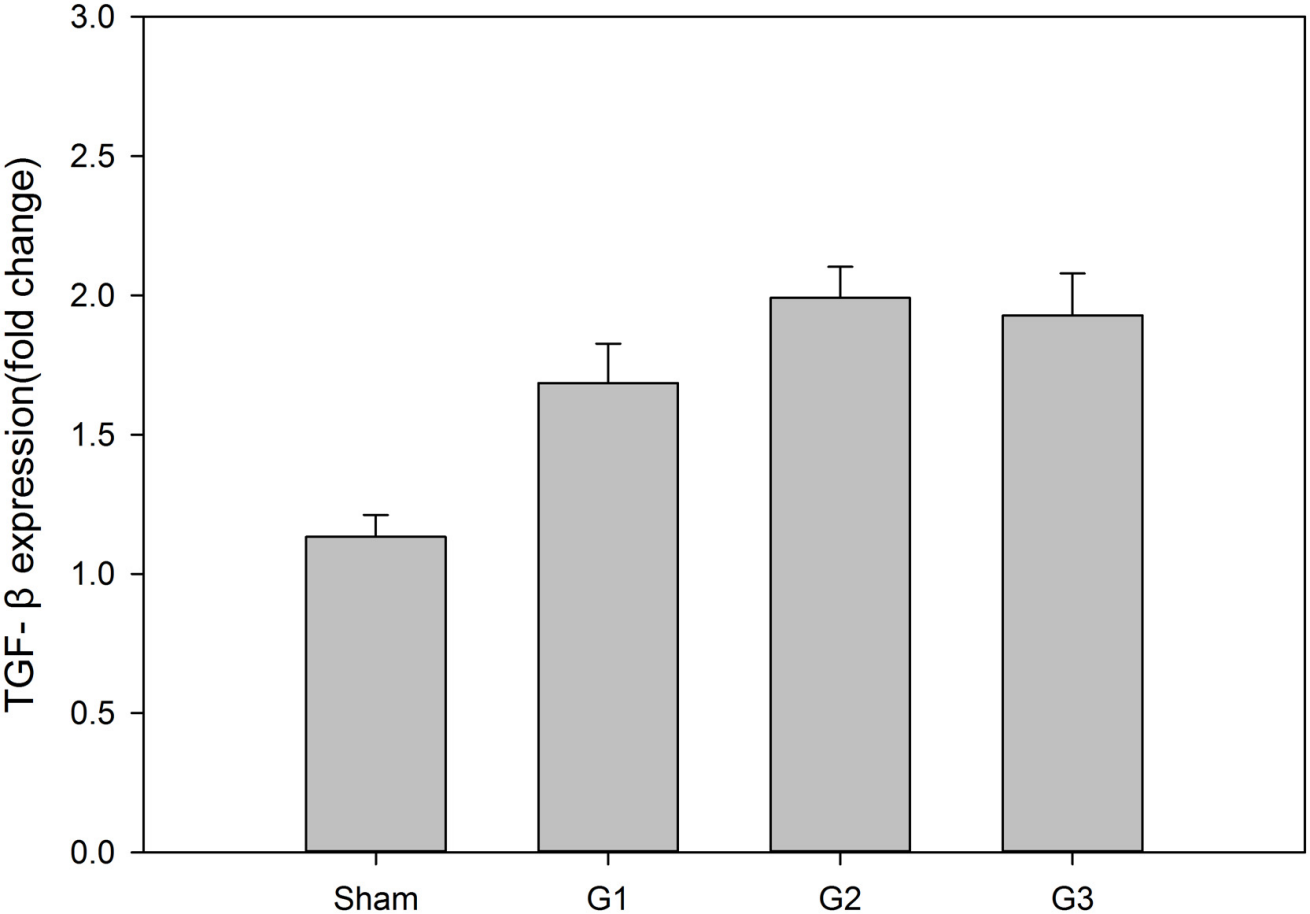
TGF-β expression at 7 days after implantation. RT-PCR was used to evaluate TGF-β expression at 7 days after implantation. The means + SEM of six mice are shown.

TGF-β, as a regulating factor, is present in non-parenchymal liver cells when the liver is damaged and leads to a modulating fibroblast phenotype and gene expression [12]. TGF-β levels were significantly different among the four groups (p = 0.000); however, there were no apparent differences (p = 0.213) among G1, G2, and G3 or between G2 and G3 (p = 0.723), as shown in Fig 7.

### Apoptosis

**Fig 8:**
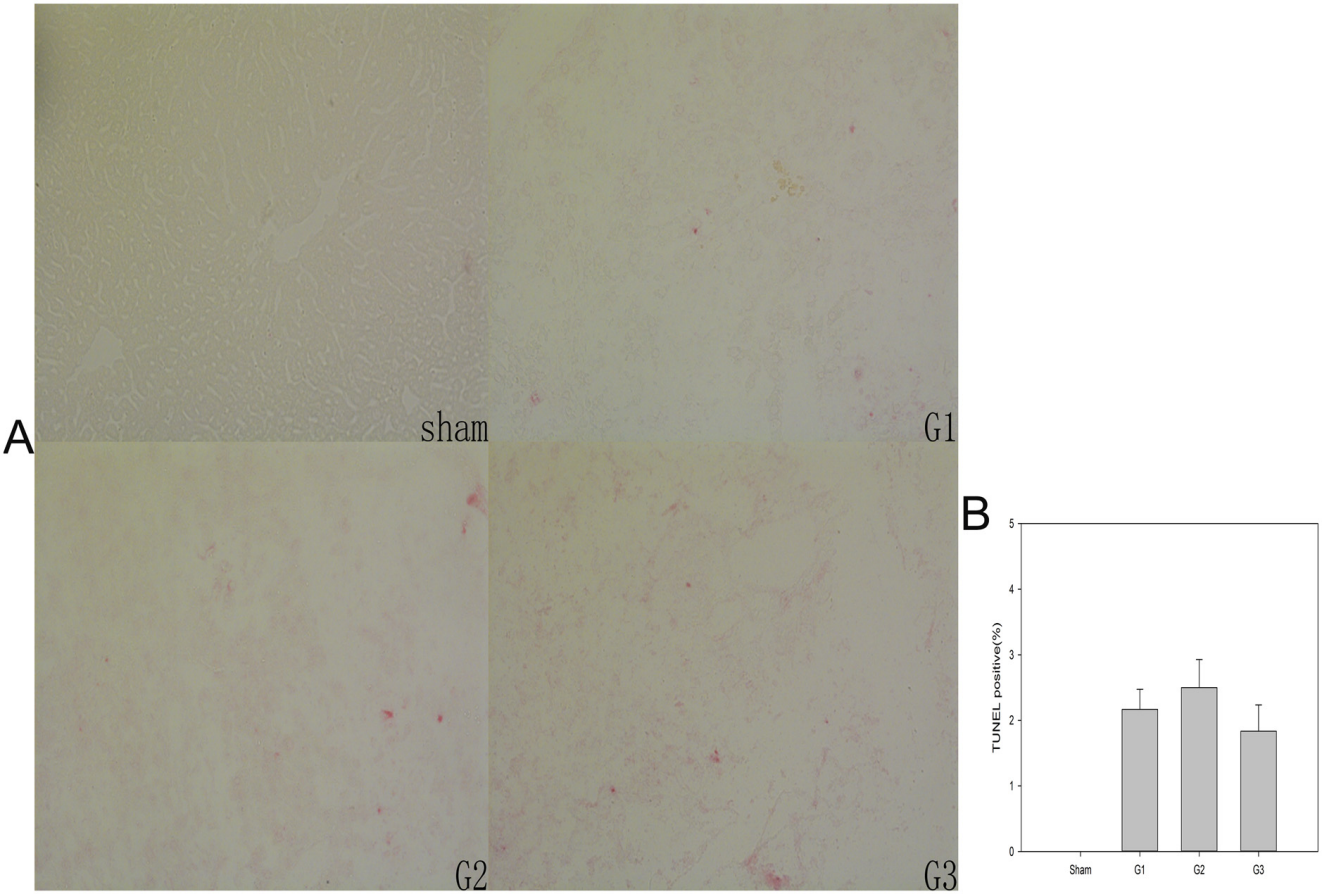
TUNEL staining of the liver grafts at 7 days after implantation. Apoptotic cells were detected by immunohistochemical labeling of DNA strand breaks. Ten fields were captured at random under a 10× objective lens. TUNEL-positive and -negative cells were quantified. The means + SEM of six mice are shown.

As another marker of liver injury, apoptosis was evaluated by TUNEL. TUNEL staining revealed no differences (p = 0.485) in apoptosis among the three groups at 7 days after transplantation. However, the percentage of TUNEL-positive cells in G3 was 1.8%, which was lower than that in G2, as shown in Fig 8.

## Discussion

OLT in the mouse is one of the most challenging procedures conducted in experimental surgeries. This model has provided many advantages for biomedical research investigating liver issues, such as liver transplantation, transplantation immunology, and ischemia reperfusion injury, among others [13–15]. However, the establishment of a stable mouse model of OLT is not an easy task. In clinical liver transplantation, reconstruction of the hepatic artery is mandatory; however, early thrombosis of the hepatic artery results in graft failure and death if retransplantation is not implemented as quickly as possible. The liver has a dual blood supply from the hepatic artery and portal vein (PV), which provide approximately 25% and 75% of the blood flow to the liver, respectively. More importantly, the hepatic artery flow provides approximately 50% of the oxygen delivered to the liver, which is crucial for alleviating injury to the bile ducts and hepatocytes [16]. When Qian et al performed non-arterialized orthotopic mouse liver transplantation, moderate fibrosis, ductile proliferation, and mild lymphocytic infiltration were discovered [4]. However, Steger et al did not find similar characteristics when using an arterialized orthotopic liver transplantation model in mice [17]. Furthermore, the AOLT model more closely mimics human OLT than the model without reconstruction of the hepatic artery, and therefore, it is widely accepted by clinicians and researchers. Although reconstruction of the hepatic artery by stent or suture has been widely employed in the arterialization of orthotopic liver transplantation (AOLT) in rat models [18], the suture method of arterial reconstruction is extremely complicated. This procedure requires a skilled operator in the mouse AOLT model, and the approach of arterial reconstruction by stent is not widely promoted in this model [19]. The goal of the present study was to demonstrate whether there was an obvious difference between reconstruction of the hepatic artery by stent compared with by aortic trunk. In G2, the celiac trunk with the hepatic artery was removed along with the entire abdominal aorta below the celiac trunk. The donor and recipient abdominal aorta were end-to-side anastomosed. Although this procedure reduces the incidence of hepatic artery thrombosis, it must be performed by a skilled operator [20]. Moreover, the procedure required much more time compared with G3, extending the anhepatic time and potentially leading to failure and death. The mean time required for reconstruction of the hepatic artery was 3 min by stent (G3) and 6 min by the aortic trunk method (G2). Therefore, the anhepatic time could be reduced in the G3 group. Moreover, the serum alanine aminotransferase levels (p < 0.05) and the operation time (p < 0.05) were markedly lower in G3 compared with G2 or G1. The mean total operation time was reduced by 13.5 min in G3 compared with G2. In addition, there were no apparent differences in serum ALT levels between G2 and G3 (p = 0.09). However, the arterial reconstruction by stent approach was able to reduce the incidence of hepatic artery thrombosis. The patency of the hepatic artery was verified in eight of the ten mice in G3 and in six of the ten mice in G2 at 7 d after AOLT. The stent can provide efficient high blood flow, which may interfere with the formation of thrombi. In contrast, reconstruction of the hepatic artery by the aortic trunk method may kink and potentially lead to early thrombosis. We modeled this method in the mouse to reconstruct the hepatic artery with a stent based on a technique that has been previously described in the rat [21]. Although Zhou et al used the stent to reconstruct the hepatic artery, the differences between reconstruction of the hepatic artery by the aortic trunk and by stent were not clear [7]. We confirmed that the 7-day survival rate, necrosis, apoptosis, and TGF-β levels did not differ significantly among the three groups. We generated stents made by stretching disposable epidural catheters, a technique that differs from that employed by Zhou et al. Zhou et al used Micro-Renathane implantation tubing as the arterial stent. However, Micro-Renathane implantation tubing has a high cost. Two types of arterial stents constructed of polyurethane, in contrast to the ordinary ethylene catheter, can reduce the adhesion rate of platelets and erythrocyte on the intravascular wall, which is crucial for preventing the formation of a thrombus. This method of hepatic artery reconstruction by stent has several advantages: first, it is simple and technically straightforward; second, the duration of anastomosis is reduced compared with suture anastomosis, shortening the anhepatic time [18]; third, it can reduce the incidence of hepatic artery thrombosis. Although this model also requires the expertise of a micro-surgeon, the shorter learning curve will allow for broader dissemination of the technique in the field of biomedical research. The use of a stent for arterialization for hepatic artery reconstruction in the mouse AOLT model should be limited to use in short-term follow-up experiments. Long-term experiments, including 30-d and longer survival assessments, will reveal differences in survival rates and histologic changes compared with the established sewn vascular anastomosis model.

In conclusion, reconstruction of the hepatic artery by stent can be performed quickly with a high patency rate. This approach to AOLT not only shortens both the donor and recipient surgery durations but also reduces the technical difficulty compared with previously described methods. This method has the potential to facilitate the model of AOLT in mice and be widely used in the field of biomedical research.

## Supporting Information

S1 ARRIVE Checklist All the experimental design and procedure have followed the ARRIVE guidelines for animal research. (PDF)
